# SARS-CoV-2 sublineages recovered from southern Brazilian cases during Omicron wave in 2023, early introduction of JN.1

**DOI:** 10.1016/j.bjid.2025.104557

**Published:** 2025-06-25

**Authors:** Mellanie Fontes-Dutra, Micheli Filippi, Meriane Demoliner, Alexandre Sita, Fernando Rosado Spilki

**Affiliations:** a Universidade Feevale, Laboratório de Microbiologia Molecular (LMM), Novo Hamburgo, RS, Brazil; b Universidade do Vale do Rio dos Sinos (UNISINOS), Programa de Pós-Graduação em Alimentação, Nutrição e Saúde, São Leopoldo, RS, Brazil

**Keywords:** SARS-CoV-2, Genomic surveillance, Nucleotide, Omicron

## Abstract

Since the emergence of the novel coronavirus SARS-CoV-2, public health measures have adapted as the virus evolve and acquired greater transmissibility and escape from the previous immune response. Genomic surveillance is a reliable and decisive tool for monitoring the evolutionary dynamics of the virus and its nucleotide diversity. Rio Grande do Sul is a southern Brazilian state that borders Argentina and Uruguay, and genomic and epidemiological surveillance led to early detection of COVID-19 variants, as seen in P.1 lineage. The study aimed to investigate the genetic characterization of SARS-CoV-2 Omicron sublineages in Rio Grande do Sul, Brazil, during 2023. By obtaining viral RNA from nasopharyngeal swabs positive for SARS-CoV-2, we performed high-throughput sequencing and data were analyzed using bioinformatic approaches. Our results revealed a dynamic change in Omicron sublineages during 2023, with the occurrence of JN.1+JN.1* reads during December 2023, parallel to the first JN.1 official record in Brazil, occurred in Ceará state, which is in the northeast region of Brazil. These data revealed a distinct nucleotide diversity in S gene of JN.1 reads, highlighting the importance of genomic surveillance in Rio Grande do Sul for the early detection of the entry of future SARS-CoV-2 variants into Brazil.

The emergence of the novel coronavirus SARS-CoV-2 posed challenges to genomic surveillance worldwide, due to its notorious evolutionary dynamics, high transmissibility, and, recently, immune evasion seen in a variety of strains.^1^^,^^2^ Among the Variants of Concern (VOC) classification, Omicron, which was identified firstly in 2021,^3^ has gained global predominance, giving rise to different sublineages with distinct collection of mutations, especially in the S gene, which codes Spike protein and it is target by different vaccines currently in use.^4^^,^^5^

During 2022, Omicron sublineages spread and predominated in Brazil, leading to an increase in COVID-19 cases,^6^^,^^7^ with the identification of OmicronL452R, for the first time, in Southern Brazil (Rio Grande do Sul),^8^ diversifying and surging different sublineages since then.^9^ Rio Grande do Sul is a southern Brazilian state that borders Argentina and Uruguay, which is a location of interest for genomic and epidemiological surveillance for early detection of COVID-19 variants, as seen in P.1 lineage, which was circulating in southern Brazil prior to November 2020.^10^

Therefore, the present study aimed to investigate the genetic characterization of SARS-CoV-2 Omicron sublineages in five cities of Rio Grande do Sul, Brazil, during 2023. In addition, our study contributes to a more comprehensive understanding of nucleotide diversity especially in genomes recovered and related to JN.1 sublineage, which became the current predominant Omicron sublineage in Brazil.

Nasopharyngeal swabs positive for SARS-CoV-2 of individuals in the cities of Campo Bom (70), Santa Cruz do Sul (10), Novo Hamburgo (4), Estância Velha (2), and São Leopoldo (1), in the state of Rio Grande do Sul, Brazil, were collected during January to December 2023 and submitted to High-Throughput Sequencing (HTS) in the Molecular Microbiology Laboratory (LMM) from Feevale University. This study was approved (Rede Corona-ômica BR MCTI/FINEP affiliated to RedeVírus/MCTI (FINEP = 01.20.0029.000462/20), CNPq = 404,096/2020–4) by the National Research Ethics Committee and the Institutional Ethics Review Board of Feevale University, following national and international ethical standards.

The genetic material of 87 SARS-CoV-2 positive samples was extracted with the commercial kit MagMAX™ CORE Nucleic Acid Purification Kit (Applied biosystems™, Thermo Fisher Scientific Kit, Waltham, MA, USA), using KingFisher ™ Duo Prime automated equipment (Thermo Fisher Scientific™). Reverse Transcription followed by qPCR (RT-qPCR) was performed using specific primers, as described by Corman et al.^11^ The whole viral genome library was obtained by using Illumina® COVIDSeq Kit, as previously described.^12^ High-throughput sequencing was implemented using the Illumina MiSeq platform using MiSeq Reagent Kit v3 (600 cycle) from Illumina Inc. (Foster City, CA, USA).

The FASTq reads were aligned with complete SARS-CoV-2 genomes using Geneious Prime Software and Multiple Alignment program for amino acid or nucleotide sequences (MAFFT), version 7. SARS-CoV-2 variants were characterized using the NextClade online platform and sublineages were identified by Pangolin online platform (https://github.com/hCoV-2019/pangolin). The phylogenetic tree was obtained by using MEGA X software (11.0.13 version), using the Maximum Likelihood model (Tamura-Nei model) and non-uniformity of evolutionary rates among sites was modeled using discrete Gamma Distribution with five rates categories. Nucleotide diversity was analyzed using workflows “Map Reads then Find Variations/SNP” from Geneious Prime Software. All sequences were deposited and are available in the international GISAID database (Supplementary Table 2).

Genomic sequencing of SARS-CoV-2 is an essential tool for tracking the evolution of the virus,^13^ providing a detailed genetic characterization of circulating variants, helping to monitor viral dynamics and to determine public health strategies. The genetic characterization revealed different Omicron sublineages (22B, 22E, 22F, 23A, 23E, 23 G ‒ Supplementary Table 1) in samples, as demonstrated in the inferred phylogenetic tree (Fig. 1).

**Fig. 1:**
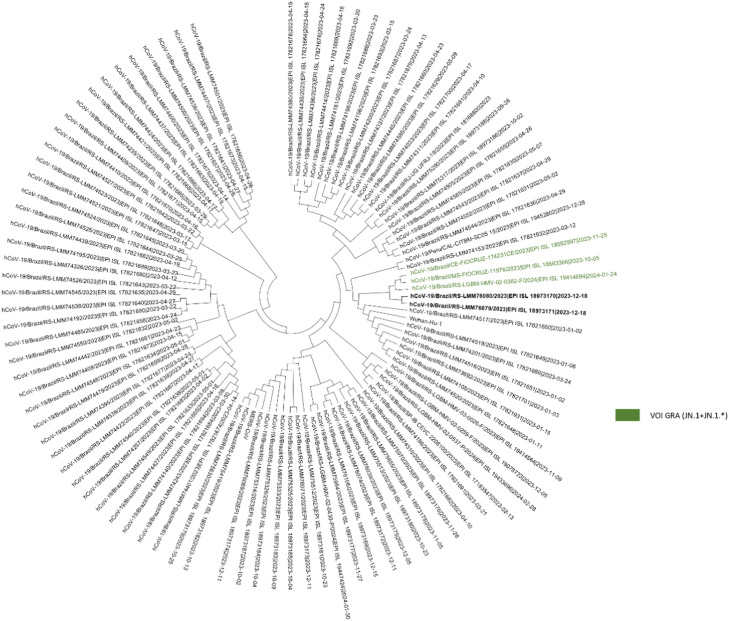
**Phylogenetic tree.** The reference genome sequences characterized as Omicron VOI GRA (JN.1 + JN.1*) are green and the genomes recovered from the sequencing of samples from Rio Grande do Sul are highlighted in bold font.

Since January 2021, LMM has been conducting epidemiological surveillance of SARS-CoV-2. Following the emergence of the Omicron variant (comprising several sublineages) in December 2021, it was possible to demonstrate that this variant has been predominant since January 2022, mirroring the global scenario. During 2023, the present data revealed an exclusive predominance of Omicron and its sublineages (Fig. 2). In January, the predominant sublineage was BQ1.1 (67 %), with other sublineages such as BE10 (17 %) and BA4.6 (17 %) present in smaller proportions. March showed greater diversity of sublineages, including XBB1.5 (22 %), XBB2.3 (22 %), FE1.1 (17 %) and XBB1.5.13 (11 %). In April, XBB.1.5.86 (33 %) predominated, with other sublineages such as XBB.2.3 (28 %) and XBB.1.5 (19 %) having fewer sequences. In May, XBB.1.5 (29 %) and XBB.2.3 (29 %) became the most frequent sublineage.

**Fig. 2:**
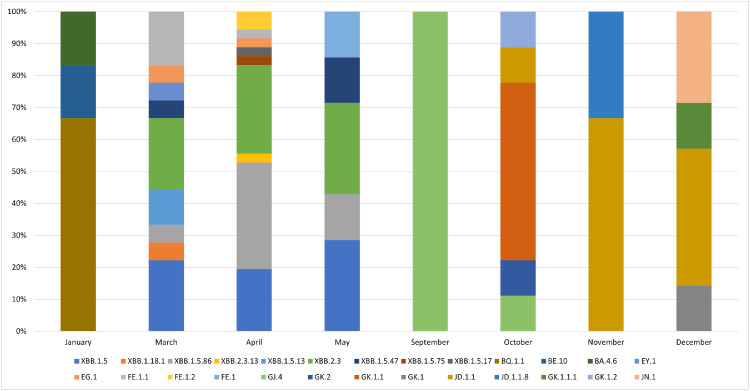
**Sublineage Frequency.** Distribution of SARS-CoV-2 sub lineages sequenced by the Molecular Microbiology Laboratory at Universidade Feevale during 2023. Y-axis: percentage ( %) of sequences of each sublineage within the dataset analyzed for each month. X-axis: represents the months of 2023 in which the sequences are distributed. Stacked Bars: each color within the bar corresponds to a specific SARS-CoV-2 sublineage (the height of each colored segment within a bar indicates the proportion of that specific sublineage relative to the total sequences analyzed for that month).

In September, GJ.4 (100 %) was the predominant sublineage. In October, the predominant sublineage shifted again to GK.1.1 (56 %), with other sublineages emerging, such as JD.1.1 (11 %) and GK.1.2 (11 %), according to GISAID. November presented the predominance of JD.1.1 (67 %), followed by JD.1.1.8 (33 %). Finally, in December, the predominant sublineages were JD.1.1 (43 %), followed by JN.1 (29 %), GK.1.1.1 (14 %), and GK.1 (14 %).

The dynamics of SARS-CoV-2 sublineages throughout the year can be observed in Fig. 2, highlighting how different variants emerge, predominate, and are eventually replaced by others. This information is crucial for epidemiological surveillance and informing public health strategies, such as vaccination and other infection control measures.^14^

Additionally, during December 2023, the Health and Environmental Surveillance Secretariat (SVSA) of Health Ministry highlighted the presence of JN.1 strain, derived from BA.2.86, detected in Ceará, northwest Brazil (technical report n° 83/2023-CGVDI/DPNI/SVSA/MS). The present data revealed the circulation of JN.1 strain in Rio Grande do Sul during December 2023. The phylogenetic tree revealed a closer relationship among JN.1+JN.1* reference genome sequences and the *reads* obtained in the study, especially considering the JN.1 genome sequence detected in Ceará, Brazil, suggesting that JN.1 might be circulating in Rio Grande do Sul as early as in Ceará. Besides, the nucleotide variation in Spike (S) gene revealed a collection of non-defining mutations in JN.1 *reads,* evidencing the continuous evolutionary dynamics of the SARS-CoV-2 genome over different subpopulations. Genomic regions with high nucleotide diversity, as indicated by peaks in the graph in Supplementary Fig. 1 (a‒b), may reflect points of selective pressure where the virus is rapidly adapting to changes in the host environment, which must be investigated in further studies.

Considering the end of the 2023 period, the data available on the SIVEP-Gripe public access platform shows an increase in cases of Severe Acute Respiratory Syndrome (SARS) and in the number of deaths in Rio Grande do Sul, beginning in September and peaking in November 2023, followed by a drop in the following period.

Unfortunately, we don't have specific data available for the municipalities where the individuals included in the samples lived, only for the state of Rio Grande do Sul. However, these data show that the detection of JN.1 in December in our sample was not parallel to the increase in SARS cases or deaths in the state during this period. This may be due to the partial immunity obtained by vaccination and/or hybrid immunity of the population, but also because the effect may have been greater at the beginning of 2024, after the New Year's Eve festivities, where there is a tendency for cases to increase after this period. It is important to consider the limitations of this detection data, as the number of sequences is low, and the variant may have been underreported at the beginning of the introduction.

The data obtained reflect the global trend of the predominance of the Omicron variant and its sublineages throughout 2023. The present study pointed that XBB strains, such as XBB.1.5 and XBB2.3 sublineagess have been identified worldwide as one of the predominant sublineages due to its ability to evade immune response and increase transmissibility. The constant evolution and emergence of new sublineages, such as JN.1 strain, highlights the ongoing need for genomic surveillance to understand viral dynamics and adapt public health strategies as needed. Compared with data from the rest of Rio Grande do Sul, similar trends in variants are observed. The predominance of the Omicron variant and its sublineages is consistent both locally and regionally, reflecting the global epidemiological scenario.

## Conflicts of interest

The authors declare no have conflicts of interest.

## Appendix Supplementary materials

Image, application 1
