# Supplementary material for: SARS-CoV-2 sublineages recovered from southern Brazilian cases during Omicron wave in 2023, early introduction of JN.1
Source: Braz J Infect Dis. 2025 Jun 25;29(4):104557. doi: 10.1016/j.bjid.2025.104557 (PMC12246843; doi:10.1016/j.bjid.2025.104557)

**BJID-D-24-00385_Supplementary Material**

**Supplementary Table 1**

| **SARS-CoV-2 Variant** | **ID GISAID Genome** |
| --- | --- |
| VOI GRA (XBB.1.5+XBB.1.5.*) | hCoV-19/Brazil/RS-LGBM-HMV-02-0430-P/2024\|EPI ISL 19447424\|2024-01-30 |
|  | hCoV-19/Peru/CAL-CITBM-SC05 15/2023\|EPI ISL 19452802\|2023-12-28 |
| VOI GRA (EG.5+EG.5.*) | hCoV-19/Brazil/RS-LGBM-HMV-02-0537-P/2024\|EPI ISL 19433696\|2024-02-28 |
|  | hCoV-19/Brazil/RS-LGBM-HMV-02-0259-F/2023\|EPI ISL 19078723\|2023-12-05 |
| VOI GRA (XBB.1.16+XBB.1.16.*) | hCoV-19/Brazil/RS-LGBM-HMV-03-0026-F/2023\|EPI ISL 19414564\|2023-11-09 |
| VOI GRA (JN.1+JN.1.*) | hCoV-19/Brazil/RS-LGBM-HMV-02-0362-F/2024\|EPI ISL 19414694\|2024-01-24 |
|  | hCoV-19/Brazil/MS-FIOCRUZ-11976/2023\|EPI ISL 18563366\|2023-10-05 |
|  | hCoV-19/Brazil/CE-FIOCRUZ-174231CE/2023\|EPI ISL 18592997\|2023-11-25 |
| VUM GRA (XBB.1.9.2+XBB1.9.2.*) | hCoV-19/Brazil/SP-IB CEVC 2206100/2023\|EPI ISL 17183547\|2023-02-13 |
| VUM GRA (XBB.2.3+XBB.2.3.*) | hCoV-19/Brazil/RJ-UG UFRJ-19/2023\|EPI ISL 18168800\|2023 |
| MERS-CoV | MERS-CoV (MH734115.1) |
| Wuhan-Hu-1 | Wuhan-Hu-1 (NC 045512) |

**Supplementary Table 2**

| **Virus name** | **City** | **Accession ID** | **Lineage GISAID** | **Pango lineage (Nextclade)** | **Collection date** | **Gender** | **Patient age** | **Clade** |
| --- | --- | --- | --- | --- | --- | --- | --- | --- |
| hCoV-19/Brazil/RS-LMM74520/2023 | Santa Cruz do Sul | EPI_ISL_17821648 | BQ.1.1 | BA.5.3.1.1.1.1.1.1 | 11/01/2023 | Female | 47 | GRA |
| hCoV-19/Brazil/RS-LMM74519/2023 | Santa Cruz do Sul | EPI_ISL_17821649 | BE.10 | BA.5.3.1.10 | 06/01/2023 | Female | 54 | GRA |
| hCoV-19/Brazil/RS-LMM74517/2023 | Santa Cruz do Sul | EPI_ISL_17821650 | BA.4.6 | BA.4.6 | 02/01/2023 | Female | 39 | GRA |
| hCoV-19/Brazil/RS-LMM74516/2023 | Santa Cruz do Sul | EPI_ISL_17821651 | BQ.1.1 | BA.5.3.1.1.1.1.1.1 | 02/01/2023 | Female | 49 | GRA |
| hCoV-19/Brazil/RS-LMM73692/2023 | Novo Hamburgo | EPI_ISL_17821701 | BQ.1.1 | BA.5.3.1.1.1.1.1.1 | 03/01/2023 | Female | 37 | GRA |
| hCoV-19/Brazil/RS-LMM74108/2023 | Campo Bom | EPI_ISL_17821931 | BQ.1.1 | BA.5.3.1.1.1.1.1.1 | 15/01/2023 | Male | 41 | GRA |
| hCoV-19/Brazil/RS-LMM74527/2023 | Santa Cruz do Sul | EPI_ISL_17821642 | XBB.1.5 | XBB.1.5 | 22/03/2023 | Female | 27 | GRA |
| hCoV-19/Brazil/RS-LMM74526/2023 | Santa Cruz do Sul | EPI_ISL_17821643 | XBB.1.18.1 | XBB.1.18.1 | 22/03/2023 | Female | 43 | GRA |
| hCoV-19/Brazil/RS-LMM74525/2023 | Santa Cruz do Sul | EPI_ISL_17821644 | FE.1.1 | XBB.1.18.1.1.1 | 20/03/2023 | Female | 51 | GRA |
| hCoV-19/Brazil/RS-LMM74524/2023 | Santa Cruz do Sul | EPI_ISL_17821645 | FE.1.1 | XBB.1.18.1.1.1 | 20/03/2023 | Female | 53 | GRA |
| hCoV-19/Brazil/RS-LMM74523/2023 | Santa Cruz do Sul | EPI_ISL_17821646 | XBB.1.5.13 | XBB.1.5.13 | 17/03/2023 | Female | 47 | GRA |
| hCoV-19/Brazil/RS-LMM74521/2023 | Santa Cruz do Sul | EPI_ISL_17821647 | XBB.1.5.13 | XBB.1.5.13 | 15/03/2023 | Female | 44 | GRA |
| hCoV-19/Brazil/RS-LMM74243/2023 | Campo Bom | EPI_ISL_17821684 | XBB.1.5.86 | XBB.1.5.86 | 30/03/2023 | Male | 58 | GRA |
| hCoV-19/Brazil/RS-LMM74235/2023 | Campo Bom | EPI_ISL_17821685 | XBB.1.5 | XBB.1.5 | 29/03/2023 | Male | 57 | GRA |
| hCoV-19/Brazil/RS-LMM74201/2023 | Campo Bom | EPI_ISL_17821686 | EY.1 | BA.5.3.1.1.1.1.1.1.13.1.1.1.1 | 24/03/2023 | Female | 37 | GRA |
| hCoV-19/Brazil/RS-LMM74200/2023 | Campo Bom | EPI_ISL_17821687 | XBB.2.3 | XBB.2.3 | 24/03/2023 | Male | 22 | GRA |
| hCoV-19/Brazil/RS-LMM74198/2023 | Campo Bom | EPI_ISL_17821688 | XBB.2.3 | XBB.2.3 | 23/03/2023 | Female | 62 | GRA |
| hCoV-19/Brazil/RS-LMM74195/2023 | Campo Bom | EPI_ISL_17821689 | FE.1.1 | XBB.1.18.1.1.1 | 23/03/2023 | Female | 37 | GRA |
| hCoV-19/Brazil/RS-LMM74192/2023 | Campo Bom | EPI_ISL_17821690 | XBB.1.5 | XBB.1.5 | 22/03/2023 | Female | 61 | GRA |
| hCoV-19/Brazil/RS-LMM74186/2023 | Campo Bom | EPI_ISL_17821691 | EG.1 | XBB.1.9.2.1 | 21/03/2023 | Male | 28 | GRA |
| hCoV-19/Brazil/RS-LMM74181/2023 | Campo Bom | EPI_ISL_17821692 | XBB.2.3 | XBB.2.3 | 20/03/2023 | Male | 59 | GRA |
| hCoV-19/Brazil/RS-LMM74158/2023 | Campo Bom | EPI_ISL_17821693 | XBB.2.3 | XBB.2.3 | 15/03/2023 | Female | 44 | GRA |
| hCoV-19/Brazil/RS-LMM74140/2023 | Campo Bom | EPI_ISL_17821694 | XBB.1.5.47 | XBB.1.5.86 | 08/03/2023 | Male | 68 | GRA |
| hCoV-19/Brazil/RS-LMM74153/2023 | Campo Bom | EPI_ISL_17821932 | XBB.1.5 | XBB.1.5 | 12/03/2023 | Female | 70 | GRA |
| hCoV-19/Brazil/RS-LMM74545/2023 | Campo Bom | EPI_ISL_17821635 | XBB.1.5 (consensus call) | XBB.1.5 | 29/04/2023 | Male | 77 | GRA |
| hCoV-19/Brazil/RS-LMM74544/2023 | Campo Bom | EPI_ISL_17821636 | XBB.2.3.13 | XBB.2.3.13.1 | 29/04/2023 | Male | 20 | GRA |
| hCoV-19/Brazil/RS-LMM74543/2023 | Campo Bom | EPI_ISL_17821637 | XBB.1.5.86 | XBB.1.5.86 | 29/04/2023 | Female | 23 | GRA |
| hCoV-19/Brazil/RS-LMM74539/2023 | Campo Bom | EPI_ISL_17821639 | XBB.1.5.86 | XBB.1.5.86 | 27/04/2023 | Male | 26 | GRA |
| hCoV-19/Brazil/RS-LMM74538/2023 | Campo Bom | EPI_ISL_17821640 | XBB.2.3 | XBB.2.3 | 27/04/2023 | Female | 63 | GRA |
| hCoV-19/Brazil/RS-LMM74536/2023 | Campo Bom | EPI_ISL_17821641 | XBB.1.5.86 | XBB.1.5.86 | 27/04/2023 | Female | 75 | GRA |
| hCoV-19/Brazil/RS-LMM74505/2023 | Campo Bom | EPI_ISL_17821655 | XBB.2.3 | XBB.2.3 | 26/04/2023 | Female | Unknown | GRA |
| hCoV-19/Brazil/RS-LMM74501/2023 | Campo Bom | EPI_ISL_17821656 | XBB.1.5.86 | XBB.1.5.86 | 26/04/2023 | Female | 76 | GRA |
| hCoV-19/Brazil/RS-LMM74500/2023 | Campo Bom | EPI_ISL_17821657 | EG.1 | XBB.1.9.2.1 | 26/04/2023 | Female | 17 | GRA |
| hCoV-19/Brazil/RS-LMM74485/2023 | Campo Bom | EPI_ISL_17821658 | XBB.1.5.75 | XBB.1.5.75 | 24/04/2023 | Female | 30 | GRA |
| hCoV-19/Brazil/RS-LMM74479/2023 | Novo Hamburgo | EPI_ISL_17821659 | XBB.1.5.86 | XBB.1.5.86 | 28/04/2023 | Female | 35 | GRA |
| hCoV-19/Brazil/RS-LMM74445/2023 | Campo Bom | EPI_ISL_17821660 | XBB.2.3 | XBB.2.3 | 23/04/2023 | Female | 56 | GRA |
| hCoV-19/Brazil/RS-LMM74442/2023 | Campo Bom | EPI_ISL_17821661 | XBB.1.5.86 | XBB.1.5.86 | 23/04/2023 | Male | 42 | GRA |
| hCoV-19/Brazil/RS-LMM74439/2023 | Campo Bom | EPI_ISL_17821662 | FE.1.1 | XBB.1.18.1.1.1 | 19/04/2023 | Female | 51 | GRA |
| hCoV-19/Brazil/RS-LMM74437/2023 | Campo Bom | EPI_ISL_17821663 | XBB.1.5 | XBB.1.5.107 | 19/04/2023 | Female | 57 | GRA |
| hCoV-19/Brazil/RS-LMM74430/2023 | Campo Bom | EPI_ISL_17821664 | XBB.2.3 | XBB.2.3 | 18/04/2023 | Male | 59 | GRA |
| hCoV-19/Brazil/RS-LMM74427/2023 | Campo Bom | EPI_ISL_17821665 | XBB.1.5.17 | XBB.1.5 | 18/04/2023 | Male | 26 | GRA |
| hCoV-19/Brazil/RS-LMM74424/2023 | Campo Bom | EPI_ISL_17821666 | XBB.1.5 | XBB.1.5.107.1 | 17/04/2023 | Female | 42 | GRA |
| hCoV-19/Brazil/RS-LMM74422/2023 | Campo Bom | EPI_ISL_17821667 | XBB.1.5.86 | XBB.1.5.86 | 17/04/2023 | Female | 40 | GRA |
| hCoV-19/Brazil/RS-LMM74421/2023 | Campo Bom | EPI_ISL_17821668 | XBB.1.5 | XBB.1.5 | 17/04/2023 | Female | 62 | GRA |
| hCoV-19/Brazil/RS-LMM74414/2023 | Campo Bom | EPI_ISL_17821669 | XBB.2.3 | XBB.2.3 | 16/04/2023 | Female | 43 | GRA |
| hCoV-19/Brazil/RS-LMM74410/2023 | Campo Bom | EPI_ISL_17821670 | XBB.1.5 | XBB.1.5 | 16/04/2023 | Male | 46 | GRA |
| hCoV-19/Brazil/RS-LMM74409/2023 | Campo Bom | EPI_ISL_17821671 | XBB.1.5 | XBB.1.5 | 15/04/2023 | Male | 70 | GRA |
| hCoV-19/Brazil/RS-LMM74408/2023 | Campo Bom | EPI_ISL_17821672 | XBB.1.5 | XBB.1.5 | 15/04/2023 | Female | 70 | GRA |
| hCoV-19/Brazil/RS-LMM74407/2023 | Campo Bom | EPI_ISL_17821673 | XBB.1.5.86 | XBB.1.5.86 | 15/04/2023 | Female | 30 | GRA |
| hCoV-19/Brazil/RS-LMM74401/2023 | Campo Bom | EPI_ISL_17821674 | XBB.1.5.86 | XBB.1.5.86 | 14/04/2023 | Female | 49 | GRA |
| hCoV-19/Brazil/RS-LMM74400/2023 | Campo Bom | EPI_ISL_17821675 | XBB.1.5.86 | XBB.1.5.86 | 14/04/2023 | Male | 52 | GRA |
| hCoV-19/Brazil/RS-LMM74396/2023 | Campo Bom | EPI_ISL_17821676 | XBB.2.3 | XBB.2.3 | 24/04/2023 | Female | 43 | GRA |
| hCoV-19/Brazil/RS-LMM74395/2023 | Campo Bom | EPI_ISL_17821677 | XBB.1.5.86 | XBB.1.5.86 | 24/04/2023 | Female | 35 | GRA |
| hCoV-19/Brazil/RS-LMM74380/2023 | Estância Velha | EPI_ISL_17821678 | XBB.2.3 | XBB.2.3 | 19/04/2023 | Male | 36 | GRA |
| hCoV-19/Brazil/RS-LMM74337/2023 | Campo Bom | EPI_ISL_17821679 | XBB.2.3 | XBB.2.3 | 13/04/2023 | Female | 41 | GRA |
| hCoV-19/Brazil/RS-LMM74326/2023 | Campo Bom | EPI_ISL_17821680 | FE.1.2 | XBB.1.18.1.1.2 | 12/04/2023 | Female | 26 | GRA |
| hCoV-19/Brazil/RS-LMM74311/2023 | Campo Bom | EPI_ISL_17821681 | XBB.2.3 | XBB.2.3 | 10/04/2023 | Female | 62 | GRA |
| hCoV-19/Brazil/RS-LMM74310/2023 | Campo Bom | EPI_ISL_17821682 | FE.1.2 | XBB.1.18.1.1.2 | 10/04/2023 | Female | 57 | GRA |
| hCoV-19/Brazil/RS-LMM74257/2023 | Campo Bom | EPI_ISL_17821683 | XBB.1.5.86 | XBB.1.5.86 | 02/04/2023 | Female | 31 | GRA |
| hCoV-19/Brazil/RS-LMM74033/2023 | Campo Bom | EPI_ISL_17821700 | XBB.2.3 | XBB.2.3 | 17/04/2023 | Male | 49 | GRA |
| hCoV-19/Brazil/RS-LMM74585/2023 | Estância Velha | EPI_ISL_17821629 | XBB.2.3 | XBB.2.3 | 09/05/2023 | Female | 26 | GRA |
| hCoV-19/Brazil/RS-LMM74565/2023 | Campo Bom | EPI_ISL_17821630 | FE.1 | XBB.1.18.1.1.2 | 07/05/2023 | Female | 42 | GRA |
| hCoV-19/Brazil/RS-LMM74552/2023 | Campo Bom | EPI_ISL_17821631 | XBB.2.3 | XBB.2.3 | 02/05/2023 | Female | 42 | GRA |
| hCoV-19/Brazil/RS-LMM74550/2023 | Campo Bom | EPI_ISL_17821632 | XBB.1.5 | XBB.1.5 | 02/05/2023 | Male | 28 | GRA |
| hCoV-19/Brazil/RS-LMM74549/2023 | Campo Bom | EPI_ISL_17821633 | XBB.1.5.86 | XBB.1.5.86 | 01/05/2023 | Male | 86 | GRA |
| hCoV-19/Brazil/RS-LMM74548/2023 | Campo Bom | EPI_ISL_17821634 | XBB.1.5 | XBB.1.5 | 01/05/2023 | Female | 58 | GRA |
| hCoV-19/Brazil/RS-LMM74540/2023 | Campo Bom | EPI_ISL_17821638 | XBB.1.5.47 | XBB.1.5.86 | 01/05/2023 | Male | 56 | GRA |
| hCoV-19/Brazil/RS-LMM75290/2023 | Campo Bom | EPI_ISL_18973188 | GJ.4 | XBB.2.3.3.4 | 28/09/2023 | Female | Unknown | GRA |
| hCoV-19/Brazil/RS-LMM75630/2023 | Campo Bom | EPI_ISL_18973179 | GK.1.1 | XBB.1.5.70.1.1 | 25/10/2023 | Female | Unknown | G |
| hCoV-19/Brazil/RS-LMM75513/2023 | Campo Bom | EPI_ISL_18973180 | JD.1.1 | XBB.1.5.102.1.1.8 | 23/10/2023 | Male | Unknown | GRA |
| hCoV-19/Brazil/RS-LMM75512/2023 | Campo Bom | EPI_ISL_18973181 | GK.2 | XBB.1.5.70.2 | 23/10/2023 | Female | Unknown | GRA |
| hCoV-19/Brazil/RS-LMM75419/2023 | Campo Bom | EPI_ISL_18973182 | GK.1.1 | XBB.1.5.70.1.1 | 13/10/2023 | Female | Unknown | G |
| hCoV-19/Brazil/RS-LMM75333/2023 | São Leopoldo | EPI_ISL_18973183 | GK.1.1 | XBB.1.5.70.1.1 | 09/10/2023 | Female | Unknown | GRA |
| hCoV-19/Brazil/RS-LMM75326/2023 | Campo Bom | EPI_ISL_18973184 | GK.1.1 | XBB.1.5.70.1.1 | 04/10/2023 | Female | Unknown | GRA |
| hCoV-19/Brazil/RS-LMM75325/2023 | Campo Bom | EPI_ISL_18973185 | GK.1.2 | XBB.1.5.70.1.2 | 04/10/2023 | Female | Unknown | G |
| hCoV-19/Brazil/RS-LMM75317/2023 | Campo Bom | EPI_ISL_18973186 | GJ.4 | XBB.2.3.3.4 | 02/10/2023 | Female | Unknown | GRA |
| hCoV-19/Brazil/RS-LMM75314/2023 | Campo Bom | EPI_ISL_18973187 | GK.1.1 | XBB.1.5.70.1.1 | 02/10/2023 | Female | Unknown | G |
| hCoV-19/Brazil/RS-LMM75970/2023 | Campo Bom | EPI_ISL_18973176 | JD.1.1.8 | XBB.1.5.102.1.1.8 | 28/11/2023 | Female | Unknown | GR |
| hCoV-19/Brazil/RS-LMM75964/2023 | Campo Bom | EPI_ISL_18973177 | JD.1.1 | XBB.1.5.102.1.1 | 27/11/2023 | Female | Unknown | GRA |
| hCoV-19/Brazil/RS-LMM75655/2023 | Campo Bom | EPI_ISL_18973178 | JD.1.1 | XBB.1.5.102.1.1 | 05/11/2023 | Female | Unknown | G |
| hCoV-19/Brazil/RS-LMM76106/2023 | Campo Bom | EPI_ISL_18973169 | JD.1.1 | XBB.1.5.102.1.1 | 15/12/2023 | Male | Unknown | GR |
| hCoV-19/Brazil/RS-LMM76080/2023 | Novo Hamburgo | EPI_ISL_18973170 | JN.1 | BA.2.86.1.1 | 18/12/2023 | Female | Unknown | GRA |
| hCoV-19/Brazil/RS-LMM76079/2023 | Novo Hamburgo | EPI_ISL_18973171 | JN.1 | BA.2.86.1.1 | 18/12/2023 | Male | Unknown | GRA |
| hCoV-19/Brazil/RS-LMM76074/2023 | Campo Bom | EPI_ISL_18973172 | JD.1.1 | XBB.1.5.102.1.1 | 11/12/2023 | Female | Unknown | GRA |
| hCoV-19/Brazil/RS-LMM76071/2023 | Campo Bom | EPI_ISL_18973173 | GK.1 | XBB.1.5.70.1.8 | 11/12/2023 | Female | Unknown | GRA |
| hCoV-19/Brazil/RS-LMM76069/2023 | Campo Bom | EPI_ISL_18973174 | GK.1.1.1 | XBB.1.5.70.1.1.1 | 11/12/2023 | Male | Unknown | GR |
| hCoV-19/Brazil/RS-LMM74565/2024 | Campo Bom | EPI_ISL_17821630 | FE.2 | XBB.1.18.1.1.3 | 07/05/2023 | Female | 69,6 | GRA |

**Supplementary Figure 1 Nucleotide Variations of the JN.1 Genomes.** (a‒b) Y-axis: Shows the nucleotide variations according to the genome position of *reads* obtained from JN.1 genomes. Xaxis: Represents the coverage along the genome. Peaks in the graph: Indicate regions with higher nucleotide diversity. Genome Annotation (colored bars below the graph): shows coding regions (ORFs) and other important genomic regions, such as ORF1a, ORF1b, S, M, N. (c) Mutations identified alongside S gene from JN.1 *reads* recovered from Next-Generation Sequencing.


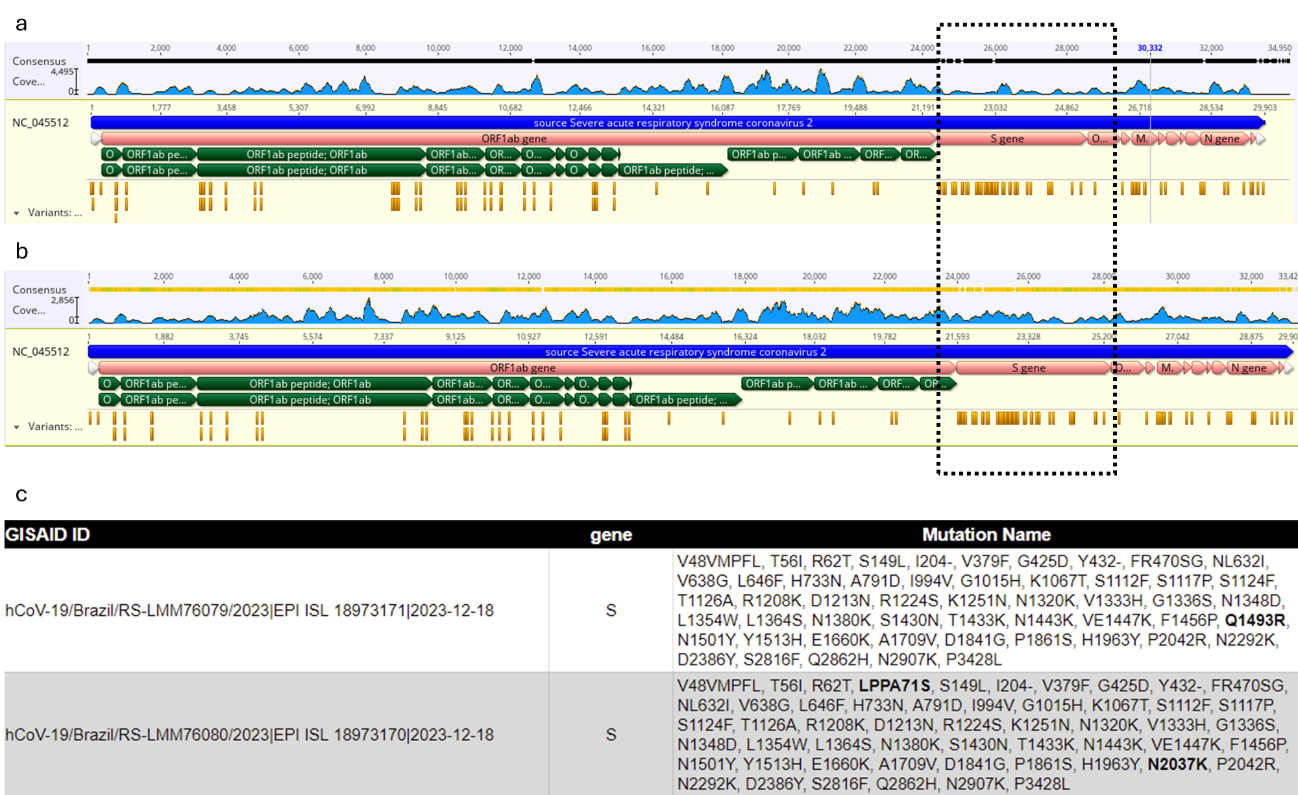

Supplement: Supplementary file 1 [file mmc1.docx]
